# Health-Promoting Compounds in Pigmented Thai and Wild Rice

**DOI:** 10.3390/foods6010009

**Published:** 2017-01-20

**Authors:** Valentina Melini, Rita Acquistucci

**Affiliations:** Consiglio per la Ricerca in Agricoltura e l’analisi dell’economia Agraria—Centro di Ricerca CREA-Alimenti e Nutrizione, Via Ardeatina 546, I-00178 Rome, Italy; rita.acquistucci@crea.gov.it

**Keywords:** pigmented rice, Thai rice, wild rice, carotenoids, phenolic compounds, anthocyanins

## Abstract

Organic pigmented Thai rice and wild rice are commonly available in specialized Italian markets and food shops, and they are gaining popularity among consumers demanding healthy foods. Indeed, the typical colour of kernels, which is the unique characteristic of pigmented cereals, is due to the accumulation of pigments that are also responsible for a number of healthy effects. The aim of this study was to provide a portrait of two organic pigmented Thai rice varieties from Thailand and one wild rice variety from Canada, imported into Italy and at Italian consumers’ disposal. To this end, the proximate composition and the content of health-promoting compounds, such as carotenoids, anthocyanins and phenolic compounds, were determined in Thai and wild rice. Moreover, the effect of cooking on phytochemicals was assessed, in order to provide reliable data on the dietary intake of bioactive compounds by samples under investigation. Results show that studied samples have a content of phytochemicals higher than white rice and comparable to other cereals. The cooking process determined a decrease of bioactive compounds in all varieties under investigation. However, some samples were found more resistant to cooking stress, and some phytochemicals were little affected by this process. Therefore, pigmented Thai and wild rice may represent a valuable source of healthy compounds and an alternative to other wholesome foods required by consumers.

## 1. Introduction

Italy is the main European rice producer [1] and Japonica varieties head the production. Some of them, such as Carnaroli, Arborio and Vialone Nano, are especially suited for making “risotto”, thanks to their quality traits, and others, such as riso Baraggia Biellese e Vercellese, Vialone Nano Veronese and Delta del Po, have been awarded Protected Designation of Origin (PDO) or Protected Geographical Indication (PGI) recognition by the European Union [2]. These varieties belong to the Italian culinary tradition and show white kernels after removal of the bran layer. 

Recently, special rice varieties, showing unique looking and exotic smell and taste, have been placed in Italian markets and can be purchased at specialized food shops. Owing to kernels with a pigmentation varying from deep-purple to brown-reddish, due to the accumulation of pigments in the pericarp and seed coat, these varieties are referred to as “pigmented rice”. Pigmented rice varieties are native to Asian countries where they were used both as foods and for therapeutic and religious purposes as well [3]. Nowadays, they are mainly grown according to organic practices by local farmers and are imported to Italy from Asian countries such as Thailand, Myanmar and Cambodia, as fair trade products.

In addition to pigmented rice, an uncommon cereal, named as “wild rice” and belonging to *Zizania* genus, has also been placed in Italian markets and has been used so far especially as a gourmet food [4]. Four species belong to the genus *Zizania*: *Zizania palustris* L., *Zizania aquatica* L., *Zizania texana* H. and *Zizania latifolia* G. The first three species are native to North America and the fourth to Asia. They look like pigmented rice due to the long and narrow cylindrical shape and the color of kernels.

Both pigmented rice and wild rice are gaining popularity among Italian consumers due to social and cultural changes in Italian population and to the increasing awareness of a need for healthy foods. As a matter of fact, pigments such as carotenoids and anthocyanins, responsible for food color, act as bioactive compounds modulating a number of physiological functions and possibly promoting health [5,6]. In addition, pigmented and wild rice is consumed as whole grain [7], so it is rich in phenolic compounds whose antioxidant activity is well documented [8].

Specialized databases of bioactive food components have been recently developed: the United States Department of Agriculture (USDA) database reporting the flavonoid content in 506 food products and the EuroFIR-BASIS, an Internet-deployed database combining food composition and biological effects data for over 300 major European plant foods, are some examples.

Because of the ever-increasing number of marketed foods, new food preparations and manufactured products, more and more efforts are required to update food composition and health-promoting compound databases. The availability of updated data is in fact critical for epidemiologists studying the role of food components and their interactions in health and diseases, and for national government agencies monitoring trends in population nutritional status through food consumption surveys, to identify the needs of nutrition education and implement appropriate strategies and interventions.

With these goals in mind, this study investigated the content of health-promoting compounds, such as carotenoids, anthocyanins, free and insoluble-bound phenolic compounds, in organic Thai and wild rice recently placed in Italian markets. While current literature is mainly focused on the detection of anthocyanins and phenols [9,10,11,12,13], this study also determined lipophilic pigment (i.e., carotenoids) content that has been poorly investigated [14,15] in pigmented and wild rice so far. In addition, the insoluble-bound fraction of phenolic compounds was considered due to its nutritional importance. Finally, as rice is always consumed after cooking, cooked samples were also analyzed and the effect of thermal process on phytochemical content was assessed, in order to have reliable evaluation of the dietary intake of healthy compounds in pigmented and wild rice.

## 2. Materials and Methods

### 2.1. Sampling

Two varieties of pigmented rice from Thailand (Thai black rice Khao Nim and Thai Jasmine red rice), certified as organic, and one variety of wild rice (*Zizania aquatica* L.) from Canada were analyzed.

Primary samples of each variety were prepared by combining at least two commercially available units, belonging to the same brand but different lot number, collected in Italian specialized shops. Subsampling was done by quartering, and samples were stored at 4 °C in the dark. Thai black rice sample was labelled as Thai black rice (BTH), Thai Jasmine red rice sample as Thai Jasmine red rice (RTH), and Canadian wild rice as Canadian wild rice (CWR).

### 2.2. Preparation of Raw and Cooked Samples for Analysis

Test samples of raw rice were prepared immediately prior to analyses by grinding rice kernels into a fine flour by means of a laboratory mill (Janke and Kunkel IKA Labortechnik, Staufen, Germany) provided with a water-cooling system to avoid oxidation. Then, the flour was passed through a 0.5 mm mesh sieve and analyses were performed.

Cooked rice samples were prepared by a cooking treatment similar to risotto preparation. In detail, a proper amount of primary sample was placed in a glass bottle and water was added at 80 °C (rice to water ratio: 1:2 *w*/*v*). Then, bottles were placed in a water bath at 100 °C and samples were cooked until complete absorption of the liquid by kernels was achieved. Afterwards, cooked rice was cooled down and frozen before freeze-drying. Freeze-dried samples were ground into a fine flour and stored in a desiccator until analysis.

### 2.3. Chemicals

Chemicals (pyrogallol, sodium hydroxide, sodium carbonate, sodium chloride, chloride acid, Folin-Ciocalteu reagent, formic acid), solvents (methanol, acetone, ethanol, hexane, ethyl acetate) and gallic acid used in the analyses were from Carlo Erba Reagenti (Rodano (MI), Italy). Carotenoid and anthocyanin standards were from Extrasynthèse (Geney, France). Distilled water was used for preparative analysis. For High-Performance Liquid Chromatography (HPLC) analysis, all solvents were HPLC grade and water was purified by a Milli-Q system (Millipore Corp, Billerica, MA, USA).

### 2.4. Instrumentation

Ultrasound irradiation was carried out by an ultrasound bath system Elmasonic S 100 H (Elma Schmidbauer GmbH, Gottlieb-Daimler-Str. 17, 78224 Singen, Germany) operating at 37 MHz.

Spectrophotometric determinations were performed by a single beam UV/VIS Beckman DU 640 spectrophotometer (Beckman Coulter srl, Cassina de Pecchi, Milano, Italy) provided with 1 cm quartz cell.

HPLC analysis was performed by using a Varian ProStar equipment provided with a diode array detector ProStar 335 (Agilent Technologies SpA, Milano, Italy).

### 2.5. Proximate Analysis

Moisture and protein content were determined according to the International Association of Cereal Science and Technology (ICC) Standard method No. 110/1 and No. 105/2 [16], respectively. Crude protein content was determined using the factor 5.95 × N for conversion. Ash content was determined according to Association of Official Analytical Chemists (AOAC) official method 923.03 [17]. Moisture was reported as g/100 g fresh weight (f.w.), while protein and ash content were reported as g/100 g dry matter (d.m.).

### 2.6. Extraction and Determination of Carotenoids

Carotenoids were extracted and determined according to the procedure reported in Acquistucci et al. [18]. In details, a proper amount of sample (2 g) was placed in a screw-capped tube and was added with 5 mL of ethanolic pyrogallol (120 g/L), 2 mL of ethanol (95%) and 2 mL of NaCl (10 g/L). Then, KOH 600 g/L (2 mL) was added and the saponification was carried out under nitrogen for 60 min at 70 °C while mixing every 5–10 min. After the alkaline digestion was completed, tubes were cooled down and added with 15 mL of NaCl (10 g/L). Then, the suspension was extracted by using a mixture of hexane and ethyl acetate (9:1 *v*/*v*) (15 mL) until decolouration of the upper organic phase was complete. Supernatants were collected and evaporated under vacuum at 40 °C. The dried residues were dissolved in a mixture of methanol and tetrahydrofuran (MeOH:THF 95:5, *v*/*v*) (1 mL) immediately prior to the HPLC analysis. Carotenoid separation was carried out by a YMC C30 reversed-phase column (250 × 4.6 mm i.d., 5 μm; CPS analitica, Milano, Italy) and elution was obtained by a 30 min gradient. Solvent A (methanol:metilen chloride:acetonitrile:water 58:30:10.5:1.5) and solvent B (methanol 100%) were used as mobile phase, at a flow rate of 1.0 mL/min. The gradient was as follows: 50% A, 0–10 min; 100% A, 25 min and hold 5 min; 50% A, 30 min. The column was re-equilibrated between two analyses for at least 10 min with 50% solvent A. All runs were performed at 30 °C and carotenoids detected at 450 nm by photodiode array detector (PAD) ProStar 335 (Varian) operating in the range of 300–600 nm. Chromatographic peaks were identified by comparing retention times and UV/VIS spectra of samples with standard solutions. PAD response for lutein and zeaxanthin was linear within the calibration range of 0.1–10.0 µg/mL and 0.01–2.50 µg/mL, respectively, with correlation coefficients exceeding 0.999. Coefficients of variation for sample replicates were below 10%.

Galaxie Chromatography Data System software (version 1.9.302.952, Varian Inc., 2700 Mitchell Drive, Walnut Creek, CA 94598-1675, USA) was used to control the equipment and to process data. Results were expressed as µg of carotenoid per g of sample on a dry matter basis (d.m.).

### 2.7. Extraction of Free Phenolic Compounds

Free phenolic compounds (FPCs) were isolated from raw and cooked samples by a two-step extraction using aqueous methanol (50:50 *v*:*v*) acidified at pH = 2 (HCl) and a mixture of acetone and water (70:30 *v*:*v*), as reported by Arranz and Saura-Calixto [19]. Samples (0.8 g) were added with the methanolic mixture (20 mL) and extracted under magnetic stirring at 250 rpm for 1 h at 23 ± 1 °C protected from light. Then, they were centrifuged and the supernatant was removed and stored in a flat-bottomed flask. The residue was added with the acetone mixture (20 mL) and the extraction repeated under the above-mentioned conditions. Extracts were pooled and FPC content was determined according to the Folin-Ciocalteu colorimetric assay detailed in Section 2.9.

### 2.8. Extraction of Insoluble-Bound Phenolic Compounds

Insoluble-bound phenolic compounds (BPCs) were extracted from the residue obtained from FPC extraction.

In details, the pellet was washed by using distilled water (50 mL), as reported by Paiva et al. [20], and it was dried at 30 °C for 8 h. The dry residue was stored at 4 °C until hydrolysis. The hydrolytic treatment was performed on 0.500 g of sample by using sodium hydroxide 2 M (10 mL) [10,21] under ultrasonic irradiation at 40 °C [22]. The alkaline hydrolysates were added with HCl until pH = 2, and then BPCs were extracted by ethyl acetate (30 mL). After centrifugation, the organic solvent was removed and the extraction repeated. Extracts were pooled and the solvent was evaporated under vacuum. The dried extract was reconstituted with 1.2 mL of MeOH:H_2_O 50:50 (*v*/*v*) immediately prior to the Folin-Ciocalteu colorimetric assay detailed in Section 2.9.

### 2.9. Determination of Total Free and Insoluble-Bound Phenolic Compound Content

The quantification of FPCs and BPCs was performed by the Folin-Ciocalteu colorimetric method as reported by Sompong et al. [10] with minor modifications. In details, a proper amount of phenolic extract (1.2 mL) was placed in a 25 mL volumetric flask and added with 6 mL of water-diluted (1:10) Folin-Ciocalteu reagent (FCR). After three minutes, sodium carbonate (Na_2_CO_3_) 75 g/L was added to adjust pH at 10–10.5 and the flask was filled to the mark with distilled water.

The absorbance of the solution was read at 753 nm after 90 min of incubation at room temperature protected from light. A blank was prepared by adding all reagents to distilled water, as described above. Gallic acid was used as a standard compound and results were expressed as mg of gallic acid equivalents (GAE) per 100 g of sample on a dry matter basis (d.m.).

### 2.10. Extraction and Determination of Anthocyanins

Anthocyanins were extracted by aqueous methanol (85:15 *v*/*v*) acidified at pH = 1.0, as reported in Kim et al. [14] with minor modifications. Briefly, a proper amount of rice (0.100–0.500 g) was added with aqueous methanol (85:15 *v*/*v*) acidified at pH = 1.0 (10 mL) and the suspension underwent ultrasound treatment for 1 min. Then, it was incubated in a water bath at 38 °C for 30 min and mixed by a vortex every 5–10 min. The suspension was centrifuged at 3000× *g*, and the supernatant was removed and stored in a flat-bottomed flask. The extract was evaporated under vacuum and the dried residue was stored at −40 °C until HPLC analysis.

Anthocyanin separation was obtained by a Phenomenex Luna^®^ 250 × 4.6 mm i.d. 5 µm column, and the elution was carried out by a 30 min gradient with water acidified by formic acid (10% *v*/*v*) (solvent A) and methanol (solvent B). The solvent gradient was programmed as follows: 5% B at 0 min, increasing to 60% within 20 min, to 100% within the next 5 min, holding at 100% for 5 min then decreasing to 5% and re-equilibrating at 5%. The solvent flow rate was set at 0.8 mL/min and the column temperature at 35 °C.

Anthocyanins were detected at 525 nm and identification was done by comparing the retention time and the UV spectra of peak samples with those of pure substances. Peak areas were used for all calculations, and results were expressed as µg of anthocyanins per g of sample on a dry matter basis (d.m.).

PAD response for cyanidin-3-*O*-glucoside (C3G) and peonidin-3-*O*-glucoside (P3G) was linear within the calibration range of 1.5–15.0 µg/mL and 0.1–1.0 µg/mL, respectively, with correlation coefficients exceeding 0.999. Coefficients of variation for sample replicates were below 10%. Galaxie Chromatography Data System software (version 1.9.302.952, Varian Inc., 2700 Mitchell Drive, Walnut Creek, CA 94598-1675, USA) was used to control the equipment and to process data.

### 2.11. Statistical Analysis

Results are reported as the mean ± SD for at least triplicate analyses of the same extract. Each sample was extracted at least twice. Tukey’s HSD (Honest Significant Difference) test was used in conjunction with ANOVA (ANalysis Of VAriance). A value of *p* < 0.05 was considered statistically significant.

## 3. Results and Discussion

### 3.1. Proximate Composition of Raw Samples

The proximate composition of raw samples under investigation is shown in Table 1. Moisture content ranged between 10.3% and 13.3%, and protein content between 9.6 and 14.0 mg/100 g (d.m.). No significant (*p* < 0.05) differences in protein content were found between BTH and RTH samples. These values were appreciably above those reported for white rice that, according to the Food and Agriculture Organization (FAO), has an average content of 6.8 g/100 g (d.m.). In CWR sample, the protein content was higher (14.0 g/100 g d.m.) than the Thai rice varieties under investigation and in keeping with Surendiran et al. [23] that report a protein concentration ranging between 10% and 18% for *Zizania* species. Such a high content was commonly found in rice bran [24], in corn [25] and in barley [25].

Ash content, referring to the mineral content of samples, ranged between 1.40 and 1.61 g/100 g (d.m.). No significant differences (*p* < 0.05) were found between BTH and CWR samples, while RTH showed the lowest amount (1.40 g/100 g d.m.). It is worth highlighting that ash content values in samples under investigation are common to brown rice [26], and the relatively high level of ash suggests that they might serve as a good source of minerals, such as potassium and phosphorus.

### 3.2. Determination of Carotenoids on Raw and Cooked Samples

The importance of carotenoids in human nutrition is due to their health effects, varying from optical enhancement within the eye [27] to immunomodulatory [28] and antioxidant functions [29]. They can be found in plants to whom they confer varied and vivid colors. On the contrary, animals and humans are not able to synthesize these molecules ex novo, therefore, they must be acquired through the diet.

The main dietary sources of carotenoids are green leafy vegetables (e.g., kale, spinach, collards, and mustard greens) and colored fruits [30], while cereals contain a relatively small amount of these molecules [20]. Despite that, the daily consumption of cereal-based products makes grains a source of carotenoids which is worth paying attention to [31]. In cereal grains, carotenoids are almost exclusively concentrated in the bran layer, thus whole grain products are richer in carotenoids than the refined equivalents.

In Italy, rice is traditionally consumed as white rice that is obtained from brown rice by removing the bran layers, thus causing the loss of carotenoids. On the contrary, pigmented rice is consumed with its bran layer intact, thus, it might contribute to the daily intake of carotenoids through diet.

In this study, two Thai rice varieties and a wild rice sample were analyzed. Carotenoid content was determined by Reversed-Phase High-Performance Liquid Chromatography (RP-HPLC) both in raw and cooked samples, in order to have a qualitative and quantitative characterization of these rice varieties at Italian consumers’ disposal and to evaluate the effect of cooking on these bioactive pigments. The main carotenoids detected in all samples were *all-trans* lutein and *all-trans* zeaxanthin, referred to hereafter as lutein and zeaxanthin. Traces of β-carotene were found in BTH and CWR.

Lutein and zeaxanthin were detected in all samples, and the former was the main carotenoid detected, accounting for more than 90% of the total carotenoids, as reported for other cereals [32]. Table 2 shows lutein and zeaxanthin content both in raw and cooked samples. In raw rice, lutein content ranged between 0.19 µg/g (d.m.) and 1.88 µg/g (d.m.): the lowest value was detected in RTH, the highest in CWR. Values were lower than those found in some Korean varieties [14], in some cultivars grown in Southern France [15] and in a Japanese black rice [33]. The different content of carotenoids might be due to genetic differences among rice varieties [34]; however, other factors such as climate or geographic site of production, harvesting and post-harvesting handling/storage, might influence rice quantitative composition of carotenoids [6].

Despite that, it is worth noticing that lutein values found in rice under investigation are comparable to carotenoid content observed by Panfili et al. [35] and Fratianni et al. [36] in some durum wheat and barley samples. Moreover, Fratianni et al. [37] reported for artichokes, apples and apricots lutein concentrations close to those detected in rice samples. Therefore, pigmented and wild rice under investigation might contribute to carotenoid intake as some fruits and vegetables do. Compared to the latter, they also have some advantages: they can be consumed all-year long and even far away from production site thanks to their longer shelf-life versus that of fruits and vegetables.

Unlike lutein, zeaxanthin accounted for only 3%–7% of total carotenoids (calculated as the sum of lutein and zeaxanthin). No statistically different (*p* < 0.05) values were found between Thai samples, while CWR showed the greatest zeaxanthin content (0.06 µg/g d.m.). In some French pigmented rice varieties, Pereira-Caro et al. [15] found zeaxanthin values higher than those detected in samples under investigation here. On the other hand, detected values were comparable to those found in French brown rice [15]. Despite their relatively low values, Thai rice samples might still be a good source of zeaxanthin. As a matter of fact, O’Connell, Ryan and O’Brien [38] observed, in a bioaccessibility study on a range of fruits and vegetables, that lower carotenoid contents in samples corresponded to higher levels of bioaccessibility or transfer into micelles. Additional studies should be performed to confirm this occurrence in cereals.

While determining some favorable changes in the texture, aroma, flavor and appearance of food, cooking may modify macro- and micro-nutrient patterns of foods. In order to evaluate the effect of this process on bioactive compounds, rice samples were added with water at 80 °C and boiled until the liquid was completely absorbed, simulating the preparation of risotto, which is the most common way to consume rice in Italy. As concerns carotenoids, it is well known that during thermal treatment they may undergo non-enzymatic oxidation or *trans*- to *cis*- isomerization [6], thus a loss of carotenoids might be observed. In cooked samples under investigation, lutein content ranged between 0.08 and 0.61 µg/g (d.m.), while zeaxanthin varied between 0.01 and 0.02 µg/g (d.m.) (Table 2). In all samples, cooking caused a significant (*p* < 0.05) loss of both xanthophills. The percent loss of carotenoids was considerably high and varied among rice species: in BTH lutein loss was 44%, in RTH 57% and in CWR 67%, while zeaxanthin decrease was 35%, 47% and 67%, in BTH, RTH and CWR, respectively.

The effect of cooking on carotenoid degradation differed among varieties and might be due to differences of the enzymatic pattern: species with oxidative enzymes resistant to heat show greater loss of carotenoids. In addition, the presence of other antioxidants may preserve carotenoids from oxidation. Overall, as concerns both xanthophills, BTH was found to be the most resistant to cooking stress, followed by RTH and then CWR. Results also showed that zeaxanthin was more stable to cooking stress than lutein in Thai rice varieties, while no differences were found in wild rice.

### 3.3. Determination of FPCs and BPCs on Raw and Cooked Samples

The content of FPCs, BPCs and TPCs (Total Phenolic Compounds) in organic black Thai, organic red Thai and wild rice under investigation is shown in Table 3.

Prior to cooking, FPCs ranged between 286.6 and 547.3 mg GAE/100 g (d.m.). Significant (*p* < 0.05) differences were found between Thai samples BTH and RTH, while CWR showed a lower amount of FPCs. Data are in agreement with Min et al. [10] that found, in some pigmented rice grown in Beaumont (USA), a FPC content varying between 240 and 697 mg GAE/100 g (d.m.). For the BTH sample, phenolic content was comparable to that found in some pigmented rice from Italy [39], while RTH rice showed FPC content higher than the Italian samples aforementioned. To the best of our knowledge, there is poor information about bioactive compound content in CWR, and no data about phenolic content are available for comparison to the current results.

After cooking, a significant (*p* < 0.05) loss of FPCs was observed only in RTH sample (24%), while in BTH and CWR samples, FPC content was found to be unaffected by cooking (Table 3). A decrease of FPCs after hydrothermal treatment has been usually observed in cereals and cereal-based products: Massaretto et al. [40] report that in some rice samples from Brazil the FPC content dropped by 83% (average), and Hirawan et al. [41] observed that FPC content in both regular and whole wheat spaghetti was 48%–78% of the original content after cooking. On the other hand, some studies showed an increase in FPCs after hydrothermal treatment: Dewanto et al. [42] report an increase of FPCs and antioxidant activity in sweetcorn, while Bryngelsson et al. [43] show an increase of phenolic content in oats after hydrothermal processes such as steaming and autoclaving.

Generally speaking, the decrease of FPC content might be due to (i) movement of phenolics out of the matrix, e.g., leaching in processing water; (ii) chemical degradation; (iii) transformation into other forms and (iv) interactions with other phenolics or food components that make FPCs less extractable [44]. In this study, the decrease of FPC content is due to causes other than leaching, because the cooking procedure applied to samples under investigation ensures cooking water is re-absorbed. On the other hand, chemical degradation and transformation or interactions with other food components might be possible.

In addition to FPCs, BPCs were determined. From a nutritional point of view, BPCs are considered more important than FPCs as they might escape from upper gastrointestinal digestion and reach the colon nearly intact. In this part of the intestine, phenolic compounds may be released from the food matrix by the action of intestinal enzymes or may undergo fermentation by colonic microbiota [45]. Thus, active metabolites responsible for a number of healthy effects may be produced [46]. Values of BFCs in raw samples varied between 52.0 and 76.8 mg GAE/100 g (d.m.). CWR showed the lowest content of BPCs, while no significant (*p* < 0.05) differences were found between BTH and RTH. In cooked samples, BPC content ranged between 68.2 and 109.7 mg GAE/100 g (d.m.). The lowest value was found in BTH, while RTH and CWR samples showed no significant (*p* < 0.05) differences.

The comparison of BPC content in raw and cooked samples enables evaluation of the effect of cooking on these bioactive molecules. It is interesting to notice that in BTH no change was observed. This might be due to the protection of BPCs from oxidative degradation by other food components with antioxidant activity. On the contrary, in the other samples under investigation a significant increase of BPCs was found. Several hypotheses might explain this trend. It might be supposed that free phenolic compounds migrated with imbibed water into the endosperm, binding proteins and/or other macromolecules, forming BPCs [44,47]. This possibly happens in RTH rice where a decrease of FPCs and an increase in BPCs were observed. For wild rice, FPC content did not change after cooking, therefore, the increase of BPC content could not be due to the formation of complexes by FPCs. Indeed, it might be explained by the effect of boiling water on rice tissue: it might soften or disintegrate rice kernels thus promoting the release of BPCs from the food matrix [44]. This was also supposed by Fares et al. [48] that observed, after cooking, an increase of BPCs in durum wheat pasta enriched with debranning fractions of wheat. Moreover, the effect of cell wall-degrading enzymes, such as esterases might facilitate BPC extraction.

The percentage of FPCs and BPCs with respect to TPCs was also expressed. TPC content was calculated as the sum of FPCs and BPCs. In raw samples, FPC percentage ranged between 85% and 89%, while BPCs were between 11% and 15%. Data are in keeping with Min et al. [12] that in bran colored rice cultivars grown in USA found 11%–27% of BPCs, Massaretto et al. [40] that reported 8%–26% of BPCs in pigmented rice genotypes grown in Brazil, and Kong and Lee [49] that found 10%–11% of BPCs in Korean pigmented rice. Therefore, pigmented rice behaves differently from non-pigmented rice and other cereals where phenolic compounds are mostly in bound form [21,50]. This trend might be due to the contribution of phenolic compounds such as anthocyanins and proanthocyanidins that are peculiar to pigmented cereals and are not esterified to cellular components, thus being extracted by organic solvents. Moreover, as observed by Goufo et al. (2014) [51], the use of HCl to acidy the mixture for the extraction of free phenolic compounds might promote the release of insoluble phenolic acids.

The comparison of TPCs before and after cooking provides an overall evaluation of cooking on phenolic compounds (Table 3). TPC content was not affected by cooking in BTH and wild rice. Nevertheless, in the former the percentage of FPCs and BPCs remained almost unchanged, while in the latter the FPC percentage dropped from 85% to 74% and BPC increased from 15% to 26%. In RTH an increase of TPCs was observed, and the increase of BPCs exceeded the decrease of FPCs. It might be supposed that in this variety, the extractability of BPCs increases due to the effect of boiling water on rice tissue or to the activity of cell-degrading enzymes.

### 3.4. Determination of Anthocyanins on Raw and Cooked Samples

All sample extracts were analyzed for anthocyanin content by RP-HPLC. Standard cyanidin-3-*O*-glucoside (C3G), peonidin-3-*O*-glucoside (P3G) and malvidin chloride were used for chromatographic peak assignment by comparing retention time and UV/VIS spectra. As shown in Table 4, in BTH both C3G and P3G were detected, in CWR only C3G was determined, while in RTH no anthocyanins were found.

In BTH, C3G content was 14.2 mg/100 g (d.m.) and P3G was 9.8 mg/100 g (d.m.). Thus, the former was the main anthocyanin accounting for 59% of the total red pigments. Data are in accordance with Escribano-Bailón et al. [52] that report C3G is the main anthocyanin in pigmented rice, followed, to a minor extent, by P3G. As concerns the concentration of C3G, in a recent study, Chen et al. [9] observed in black rice cultivars grown in Japan, a great variability in values, ranging from 30.8 to 249.6 mg/100 g. Besides genetic factors, geographical and climatic conditions may affect anthocyanin content [51]. The C3G content, detected in BTH rice under investigation, was higher than reported by Abdel-Aal et al. [53] in some pigmented cereals: in blue wheat C3G content was 2.0 mg/100 g, in purple wheat 0.4 mg/100 g and in blue barley 0.1 mg/100 g.

While the occurrence of anthocyanins in black rice varieties has been reported by several authors worldwide [9,33,39,49,51,54,55,56], the presence of this class of pigments in red rice has been observed only in some varieties. Pereira-Caro et al. [13] detected 0.3 mg/100 g of C3G in a red rice cultivar from Camargue region (France), while Chen et al. [9] identified malvidin in some Japanese red rice cultivars. In the present study, no anthocyanin was detected in the RTH sample. This might be due to a lack of this pigment in the sample or possibly because anthocyanins might occur in such a low amount that methods used may be not sensitive enough for their detection. In the wild rice sample, only C3G was detected, and only a low amount (0.3 mg/100 g d.m.). A dearth of literature exists about bioactive compounds in wild rice, however Gutek et al. [57] report that C3G represents 75% of total anthocyanins in *Zizania aquatica*.

Alongside carotenoids, anthocyanins may be lost during thermal processing. As a matter of fact, they are susceptible to variations of temperature, pH, the presence of light, oxygen and other factors [58]. Moreover, due to their solubility in water, anthocyanins may undergo leaching into boiling water. In this study, cooked samples underwent analysis in order to assess the effect of cooking on anthocyanin content. In BTH extract, C3G was 24.7 mg/100 g (d.m.) and P3G was 5.3 mg/100 g (d.m.). Interestingly, a significant (*p* < 0.05) increase of C3G was observed, while P3G content decreased by 46%. The trend observed for C3G could be explained by the fact that the risotto cooking enables water to be absorbed avoiding their loss, moreover the cooking process involves changes to the structural integrity of the cellular matrix, softening the vegetable tissue, disrupting and possibly increasing pigment extraction [59]. On the contrary, Zaupa et al. [55] observed that anthocyanin content decreased by about 28% in an Italian black rice cooked as risotto. No anthocyanins were detected in cooked sample of CWR. As previously stated for RTH, this might be due either to a degradation of pigments during cooking or to a low sensitivity in the methods used. Interestingly, the C3G content found in cooked BTH was comparable or even higher than that reported in some foods, such as red grape (3.9 mg/100 g f.w.), nectarine (6.8 mg/100 g f.w.), small red bean (1.9 mg/100 g f.w.) and plum (19.0 mg/100 g f.w.) [60].

## 4. Conclusions

This study provides a portrait of Thai pigmented rice and wild rice available in Italian markets, in terms of proximate composition and health-promoting compound content. In addition to phenolic compounds and anthocyanins, that are the most investigated bioactive molecules in pigmented rice, carotenoid content has been also evaluated, thus providing a more complete profile of the antioxidant content in samples under investigation. As in other cereals, lutein was found to be the main carotenoid and anthocyanins were detected only in black samples. In contrast to other cereals, phenolic compounds were found mainly in free form.

The effect of cooking on these components was also assessed, to more accurately determine the true dietary intake of health-promoting compounds through Thai and wild rice consumption.

Results from this study also provide reliable data for inclusion in comprehensive food composition databases which include health-promoting compounds. These databases represent a further step into reliable and extensive evaluation of dietary intakes in the Italian population, assessment of diet adequacy and development of public health recommendations.

This study also highlighted that the content of bioactive compounds in samples was comparable to other foods; therefore, the Thai and wild rice varieties under investigation can contribute to increased dietary intake of phytochemicals. Finally, the study provides information about the quality of imported food products that is usually overlooked.

## Figures and Tables

**Table 1:** Proximate composition of colored Thai and wild rice.

Sample	Moisture g/100 g (%) f.w.	Protein Content g/100 g d.m.	Ash Content g/100 g d.m.
BTH	12.3 ± 0.1 ^a^	9.6 ± 0.2 ^a^	1.58 ± 0.03 ^a^
RTH	13.3 ± 0.0 ^b^	9.8 ± 0.0 ^a^	1.40 ± 0.04 ^b^
CWR	10.3 ± 0.0 ^c^	14.0 ± 0.0 ^b^	1.61 ± 0.01 ^a^

Mean values within a column superscripted by the same letter are not significantly different at *p* < 0.05. BTH, Thai black rice; RTH, Thai Jasmine red rice; CWR, Canadian wild rice; f.w., fresh weight; d.m., dry matter.

**Table 2:** Carotenoid content (µg/g d.m.) in raw and cooked Thai and wild rice samples and loss (%) due to cooking.

	Lutein			Zeaxantin		
	Raw	Cooked	Loss	Raw	Cooked	Loss
BTH	0.64 ± 0.06 ^a,A^	0.36 ± 0.04 ^a,B^	44	0.02 ± 0.00 ^a,A^	0.01 ± 0.00 ^a,B^	35
RTH	0.19 ± 0.00 ^b,A^	0.08 ± 0.01 ^b,B^	57	0.01 ± 0.00 ^a,A^	0.01 ± 0.00 ^b,B^	47
CWR	1.88 ± 0.14 ^c,A^	0.61 ± 0.09 ^c,B^	67	0.06 ± 0.01 ^b,A^	0.02 ± 0.00 ^c,B^	67

Mean values within a column superscripted by the same letter are not significantly different at *p* < 0.05. Mean values within a row in raw and cooked samples superscripted by the same capital letter are not significantly different at *p* < 0.05. BTH, Thai black rice; RTH, Thai Jasmine red rice; CWR, Canadian wild rice; d.m., dry matter.

**Table 3:** FPC, BPC and TPC content (mg GAE/100 g d.m.) in raw and cooked Thai and wild rice samples.

	FPCs		BPCs		TPCs	
	Raw	Cooked	Raw	Cooked	Raw	Cooked
BTH	422.3 ± 3.4 ^a,A^ (85%)	474.0 ± 23.7 ^a,A^ (87%)	76.8 ± 5.1 ^a,C^ (15%)	68.2 ± 2.2 ^a,C^ (13%)	499.1 ± 8.5 ^F^	542.3 ± 25.9 ^F^
RTH	547.3 ± 14.3 ^b,A^ (89%)	413.5 ± 5.1 ^b,B^ (79%)	69.5 ± 8.7 ^a,D^ (11%)	109.7 ± 6.9 ^b,E^ (21%)	616.8 ± 23.0 ^G^	523.2 ± 14.7 ^H^
CWR	286.6 ± 5.0 ^c,A^ (85%)	268.1 ± 10.9 ^c,A^ (74%)	52.0 ± 2.9 ^b,D^ (15%)	93.8 ± 8.1 ^b,E^ (26%)	338.6 ± 20.1 ^I^	361.9 ± 0.6 ^I^

Means with different small letters in a column and with different capital letters in a row are significantly different (*p* < 0.05). Percentage of phenolic compounds compared to TPC content is reported in brackets. FPCs, free phenolic compounds; BPCs, insoluble-bound phenolic compounds; TPCs, total phenolic compounds; GAE, gallic acid equivalents; BTH, Thai black rice; RTH, Thai Jasmine red rice; CWR, Canadian wild rice; d.m., dry matter.

**Table 4:** Anthocyanin content (mg/100 g d.m.) in raw and cooked Thai and wild rice samples.

	C3G		P3G	
	Raw	Cooked	Raw	Cooked
BTH	14.2 ± 1.5 ^a,A^ (59%)	24.7 ± 1.4 ^a,B^	9.8 ± 0.5 ^C^ (41%)	5.3 ± 0.3 ^D^
RTH	n.d.	n.d.	n.d.	n.d.
CWR	0.3 ± 0.0 ^b,A^	0.3 ± 0.0 ^b,A^	n.d.	n.d.

Means with different small letters in a column and with different capital letters in a row are significantly different (*p* < 0.05). Percentage of anthocyanins in brackets. C3G, cyanidin-3-*O*-glucoside; P3G, peonidin-3-*O*-glucoside; n.d., not determined; BTH, Thai black rice; RTH, Thai Jasmine red rice; CWR, Canadian wild rice; d.m., dry matter.
